# Cellular Therapeutics for Heart Failure: Focus on Mesenchymal Stem Cells

**DOI:** 10.1155/2017/9640108

**Published:** 2017-12-17

**Authors:** Amitabh C. Pandey, Jordan J. Lancaster, David T. Harris, Steven Goldman, Elizabeth Juneman

**Affiliations:** ^1^Department of Medicine, University of Arizona, Tucson, AZ, USA; ^2^Division of Cardiology, Scripps Clinic, La Jolla, CA, USA; ^3^Department of Physiology, University of Arizona, Tucson, AZ, USA; ^4^Department of Immunobiology, University of Arizona, Tucson, AZ, USA; ^5^Section of Cardiology, Southern Arizona Veterans Health Affairs System, Tucson, AZ, USA; ^6^Sarver Heart Center, University of Arizona, Tucson, AZ, USA

## Abstract

Resulting from a various etiologies, the most notable remains ischemia; heart failure (HF) manifests as the common end pathway of many cardiovascular processes and remains among the top causes for hospitalization and a major cause of morbidity and mortality worldwide. Current pharmacologic treatment for HF utilizes pharmacologic agents to control symptoms and slow further deterioration; however, on a cellular level, in a patient with progressive disease, fibrosis and cardiac remodeling can continue leading to end-stage heart failure. Cellular therapeutics have risen as the new hope for an improvement in the treatment of HF. Mesenchymal stem cells (MSCs) have gained popularity given their propensity of promoting endogenous cellular repair of a myriad of disease processes via paracrine signaling through expression of various cytokines, chemokines, and adhesion molecules resulting in activation of signal transduction pathways. While the exact mechanism remains to be completely elucidated, this remains the primary mechanism identified to date. Recently, MSCs have been incorporated as the central focus in clinical trials investigating the role how MSCs can play in the treatment of HF. In this review, we focus on the characteristics of MSCs that give them a distinct edge as cellular therapeutics and present results of clinical trials investigating MSCs in the setting of ischemic HF.

## 1. Introduction

Heart failure (HF) has become a major epidemic throughout the world. Resulting as the common end pathway for a myriad of cardiovascular disease processes, HF is the most common cause of hospital admission in patients over 65 years old, with the number of individuals having HF reaching 8 million and expected costs in the United States exceeding 40–70 billion dollars [1]. The foundation of current therapy for HF is pharmaceutical interventions. Certain subsets of patients with HF may benefit from advanced therapies including cardiac resynchronization therapy (CRT), mechanical circulatory support devices, and even transplant, which is reserved to the sickest patients. However, these measures are not without pitfalls; pharmaceutical therapies have side effects, and CRT, while advantageous, is only available to some patients [2]. Recently, there has been a push to investigate more innovative treatments for HF that aim at not only improving clinical symptoms but also improving cardiovascular pathophysiology. While pharmaceutical and device therapy can improve the pathophysiology of ischemic HF, nonischemic HF still has limited options currently. One of the leading treatments under investigation for HF is the use of mesenchymal stem cells (MSCs). Mesenchymal stem cells are multipotent adult stem cells that have been at the forefront of regenerative medicine research. Mesenchymal stem cells are unique cells that can be cultured ex vivo and utilized as cellular therapies in a variety of disease states. Currently, MSCs are being entertained as treatment modalities in cardiovascular disease states such as acute myocardial infarction, fibrosis, and heart failure. There are other cell therapies that have been explored in translational projects including those of induced pluripotent stem cells (iPSCs) and vector-based gene therapy. Here, we focus on MSCs and their desirable properties as cellular therapeutics in heart failure and implicate their potential use in clinical practice.

## 2. Mesenchymal Stem Cells in Cell-Based Therapies

**Figure 1:**
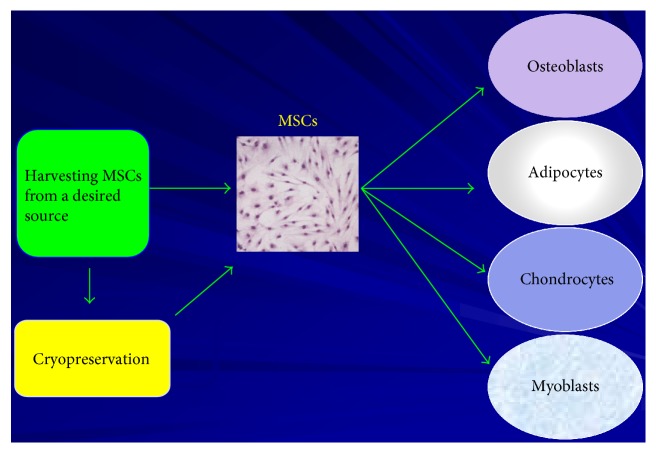
Multipotent capacity of mesenchymal stem cells. MSCs are derived from numerous tissue sources including bone marrow and adipose tissue. They are able to differentiate into various end-cell types including osteoblasts, adipocytes, chondrocytes, and myoblasts. Additionally, they are immunoprivileged, therefore allowing autologous as well as allogeneic therapeutic potential. They can also be cryopreserved, while maintaining their multipotent properties, thus allowing them to be ideal candidates for “off the shelf” cell-based therapies.

Mesenchymal stem cells are a type of adult stem cells that are multipotent cells [3, 4]. Mesenchymal stem cells maintain the ability to give rise to a number of different end-cell lineages including bone cells, adipose cells, stromal cells, muscle cells, tendon cells, and other mesenchymal cells (Figure 1) [3–5]. Mesenchymal stem cells are utilized for endogenous cell-to-cell communication and paracrine signaling and also employ these properties for cellular repair when utilized in cellular therapeutics [5]. Although not conclusively proven, mesenchymal stem cells are postulated to achieve these processes via expression of a wide spectrum of secreted factors and to a lesser extent direct end-cell differentiation for replacement of damaged cells [4, 5]. Factors that are expressed by MSCs include cytokines, chemokines, and adhesion molecules, which then regulate the activation and/or inhibition of molecular signaling pathways for endogenous cellular repair [3]. Additionally, MSCs are immunoprivileged cells given the lack of expression of major histocompatibility complex II (MHC II) complexes in their multipotent state [6]. Furthermore, MSCs have been shown to decrease inflammation and inflammatory cues as well as to promote angiogenesis [3, 5, 7]. Mesenchymal stem cells represent an ideal candidate in the emerging field of regenerative medicine [8]. These properties combined with the accessibility of MSCs from the bone marrow or adipose tissues make MSCs ideal candidates for cell- and gene-based therapies.

Since their initiation, isolation, and description by Friedenstein and colleagues from bone marrow, MSCs have been considered potential candidates for cell-based therapies [9]. Mesenchymal stem cells have been isolated from a wide variety of tissues including bone marrow tissue, adipose tissue, cardiac tissue, umbilical cord tissue, as well as those of other sites [10–15]. The therapeutic potential of MSCs makes them the ideal candidates for cell- and gene-based therapies for a number of reasons including their applicability for “off the shelf use” potential in cellular therapeutics; their immunoprivileged state; their ability to express cytokines, chemokines, and adhesion molecules; and their ability to be expanded to sufficient therapeutic quantities ex vivo [3, 7, 16]. Previous studies have shown that MSCs maintain their therapeutic potential even after cryopreservation, further adding to the list of desirable characteristics of MSCs [17, 18]. Additionally, given their immunoprivileged state, allogeneic treatment modalities could be a legitimate possibility as well for the desired therapy in heart failure patients.

## 3. MSCs and Paracrine Signaling

While MSCs can differentiate along mesenchymal lineages to produce end-cell types needed for repopulation of damaged tissues, the primary mechanism postulated by which MSCs are able to direct and facilitate endogenous cellular repair is via paracrine signaling [5, 16]. It is postulated that utilizing paracrine signaling, MSCs direct and modulate the cellular microenvironment by promoting survival and proliferation of endogenous cells, induce angiogenesis, quell inflammation, inhibit apoptosis, and recruit endogenous progenitor cells to endpoint differentiation, with an end result of functional improvement (Figure 1) [19–22]. Many studies have attempted to characterize the mechanisms by which MSCs produce a more favorable microenvironment conducive to endogenous cellular repair. It appears among the key components involved in modulating cellular signaling cascades including anti-inflammatory cues, proangiogenic signaling, and avoidance by immune surveillance [3, 4, 19, 22]. Many of the cytokines expressed by MSCs work by quelling the inflammatory signaling present in disease states including HF [5, 23, 24]. Furthermore, recent studies have shown that IL-1*β* may play a critical role in instigating the onset of HF [24]. Mesenchymal stem cells are able to secrete potent levels of interleukin 1 receptor antagonist (IL-1rn), which has been thought to be a key regulator of MSC-based therapy [5].

With MSCs available from a variety of tissues, one question that frequently arises with the proposal of MSCs as cell-based therapies is “Are MSCs taken from the varying tissues equal in directing paracrine-mediated endogenous cellular repair?” Many studies have investigated the differences in MSCs isolated from different tissue sources, and the potential of these cells remains the same despite the location of isolation [3, 25]. While MSCs possess multiple attributes desirable for the ideal cell-based therapy for HF, pitfalls do currently exist as well. Limitations as to method, timing, and dose of cells remain unknown for MSC administration in the setting of HF.

Despite the fact that MSCs can be scaled to large quantities ex vivo, limitations still exist as to delivery of an adequate number of cells given the progression and individual state of the disease for a given patient [7]. This problem seems to have been overcome with the use of allogeneic MSCs from healthy donors. Other studies have reported concerns regarding retention of injected cells over time in the heart given issues of low engraftment and limited retention of MSCs or other cell-based therapies [26]. However, given that the primary function of MSCs seems to be promotion of endogenous repair via paracrine signaling rather than direct end-cell lineage differentiation, absolute cell numbers can be deceptive. Additionally, local engraftment of cells is not entirely necessary given that paracrine signaling largely contributes to the overall function of MSCs in directing endogenous cellular repair [27, 28]. Thus, while several mechanisms have been investigated to identify the ideal route of delivery of MSCs, further work is necessary regarding optimization of delivery to the area of injury given poor understanding of how MSCs would be best utilized [29–31]. Nonetheless, advancements have been made with recent clinical trials demonstrating safety of allogeneic MSCs [32]. Additionally, many of these studies were conducted in the context of acute infarction, with significant work still needing to be initiated in the setting of HF.

## 4. Heart Failure

Heart failure is a major cause of morbidity and mortality worldwide, with greater than 5.6 million individuals afflicted in the US alone [33]. Heart failure is among the most common diagnoses for hospital admission with estimates of approximately one percent of the western world afflicted and constitutes approximately 400,000 new admissions annually [34]. It is now appreciated that the underlying cellular processes in HF are an interplay of myocardial factors, systemic factors, and local inflammation [35]. Collectively, the disarray of molecular pathways, downstream signaling, and subsequent gene expression culminates in the debilitating clinical disease state of HF. Recent studies have demonstrated that inflammatory cues play a critical role in instigating the onset of HF at the molecular level with cytokines such as interleukin 1 beta (IL-1*β*) and nuclear factor kappa B (NF-*κ*B) contributing critically to left ventricular (LV) deterioration [23, 24, 36–39].

## 5. Current Treatment of Heart Failure

Current treatment strategies in HF focus on minimizing disease morbidity, reducing hospitalizations, and prevention of mortality [33]. Despite the significant economic burden as well as morbidity and mortality stemming from HF, no promising treatment modalities to reverse the disease process currently exist. In particular, end-stage HF results in a common final pathway initiated by several signaling mechanisms that are ultimately characterized by myocardial dysfunction and cardiac remodeling. Current treatment options for HF include drug therapy, cardiac resynchronization therapy, mechanical circulatory support, and/or cardiac transplantation [40–43]. While cardiac transplantation does improve mortality and quality of life, it remains a limited therapy given the epidemiologic restriction of donor hearts available. Furthermore, an ever-increasing number of HF patients have no remaining therapies available given these restrictions [44]. Potential new therapies for HF will likely require targeted molecular therapy thus integrating local and systemic inflammation, promoting neoangiogenesis, and developing a methodology by which LV dysfunction can ideally be restored. Cellular therapeutics could allow a greater number of patients afflicted with HF to benefit from therapy than is possible via current advanced heart failure therapies, especially, given consideration that the only true treatment currently available for HF patients remains cardiac transplantation, which is prohibitive given the associated costs and limited donor hearts [7]. Indeed, cell-based therapies and regenerative medicine-directed therapies for HF would significantly change the course of disease progression and patient outcomes. Mesenchymal stem cells represent an ideal candidate in the emerging field of regenerative medicine that could be at the forefront of cell-based therapies for heart failure.

## 6. Clinical Trials and MSC-Based Therapies for Heart Failure

Recently, several clinical trials are ongoing in order to determine the safety and efficacy of MSC-based treatment for acute myocardial infarction (MI), with far fewer trials investigating the use of MSC-based therapies in the setting of HF (Table 1) [45, 46]. Most of the trials utilizing MSCs as a therapeutic option are in ischemic heart failure, and there are little data to date on the treatment in nonischemic cardiomyopathies. Indeed, MSCs have been used in clinical trials to treat both ischemic and nonischemic heart failure with both approaches showing promising results [47, 48]. While these diseases differ in terms of presence or absence of coronary artery disease, the MSCs are directed at generating new myocardium. One could speculate that MSCs would potentially be more effective in nonischemic disease because the damaged myocardium still has adequate blood supply. In the ischemic HF trials, studies have already started to show improvements in regional and global systolic and diastolic function, reversal of LV remodeling, and enhanced myocardial collateralization and coronary perfusion using the regenerative potential of MSCs [49–53]. Of note, the benefits of MSC therapy appear to be seen in the relative short term, and there remains a question as to the long-term benefits of MSC therapy as in other types of cell-based therapy. This suggests that the paracrine-mediated effects of cell-based therapy may be directly related to cell survival. Mechanistically, much remains to be elucidated as to the exact means by which MSCs achieve reversal of LV remodeling. It has been suggested that limiting inflammation coupled with deposition of extracellular matrix components deposited by MSCs may help limit the total scar size thereby decreasing LV dimensions and possibly improving diastolic function [27, 28, 54–57]. Despite these advances, further investigation into the mechanisms by which MSCs are able to facilitate these actions is warranted. Some studies suggest critical roles played by cytokines, expressed by MSCs or progenitor cells recruited by MSCs, that may be integral to cardiac recovery including insulin like growth factor 1 (IGF-1), hepatocyte growth factor (HGF), and vascular endothelial growth factor (VEGF) [58–62].

With the ever-increasing popularity of MSCs as potential cellular-based therapeutics for HF, a number of clinical trials have recently been completed and several are underway investigating the perspective roles how MSCs could play in the setting of HF events (Table 1). The majority of these trials have looked at MSCs, primarily for the reasons outlined previously [3, 5, 6, 24, 28, 30]. Several trials have attempted to investigate the use of MSCs in the setting of acute and chronic HF, with a variety of strategies on cell delivery. Initial trials focused on events closer to the time of the infarct, with cell administrations occurring in the setting of acute myocardial infarction, with MSCs being investigated in an attempt to prevent ischemic cardiomyopathy [50]. Still, other studies have looked at the therapeutic potential of MSCs in the setting of nonischemic cardiomyopathies including the setting of chemotherapy and dilated cardiomyopathies [47, 63]. However, given the healthcare burden manifested by ischemic chronic HF, later trials have looked at the treatment potential of MSCs outside the time of acute MI [64]. The addition of studies investigating the population of patients afflicted with HF remains paramount given that the disease state remains the leading reason for morbidity and mortality. Furthermore, trials investigating nonischemic HF are needed as well. On a physiologic and biochemical level, chronic ischemic HF changes the microenvironment and biochemical milieu of signaling that occurs, thus altering cardiovascular physiology [32, 65, 66]. In addition to MSCs, other cell-based therapies are also under consideration in clinical trials as potential alternatives for cell-based therapies; however, the majority of trials remain focused on MSCs given their desirable characteristics, ease of use, and accessibility [42, 67–69]. Indeed, no other cell-based therapy for HF continues to hold as much potential as MSCs for a true “off the shelf” approach that can be utilized in autologous or allogeneic modalities.

## 7. Results of Clinical Trials with MSCs

Predominantly, clinical trials have demonstrated that MSCs are safe for administration without increased risk for adverse events [18, 42–44]. Furthermore, results of the previous trials suggest that treatment with MSCs does not increase risk of posttreatment arrhythmias [42]. Additionally, studies have shown significant improvement in patient exercise tolerance [42, 44]. Investigations have also looked to see if MSCs utilized as concurrent or adjuvant therapies with existing treatment modalities for HF such as in the setting of mechanical circulatory support devices can provide benefits [70]. While many of these clinical trials have found trends towards improvement in New York Heart Association (NYHA) class, the results have not always been statistically significant or with dramatic improvements in treatment versus nontreatment groups. However, this trend seems to be changing. Recent trials have started to demonstrate findings that are statistically significant, likely due to several factors, among which is increasing sample size [42, 44]. Another factor playing a role in observed results likely lies with MSCs themselves. The route of administration has been a focal point of investigation of MSCs in HF. Initial studies were in the setting of acute myocardial infarction, and intracoronary delivery was the standard practice [71]. The limitations to delivering MSCs by the intracoronary route is the observation that the cells are rapidly washed out. Investigators have tried to increase the dwelling time in the coronary artery by delivering the cells and then occluding the coronary artery. To date, it is not clear that this approach increases the number of cells remaining in the heart. Attempts have been made to investigate alternative approaches as well. Intravenous allogeneic MSCs have been tested in a small pilot study in patients with nonischemic cardiomyopathy [72]. The authors speculate that the anti-inflammatory effects of the MSCs may be the mechanism of action because the cells were delivered intravenously. Other studies have investigated the role of endomyocardial delivery of MSCs and coronary sinus approaches as well, albeit the latter was with bone marrow aspirate and not purely MSCs [32, 47, 73, 74]. However, the route of administration remains an area that requires more investigation.

Studies have quelled previous concerns regarding safety in administration of MSCs [7, 64, 65, 69]. This has even been investigated in the setting of dilated cardiomyopathies [47]. The POSEIDON-DCM trial is a randomized comparison of allogeneic versus autologous MSCs for nonischemic dilated cardiomyopathy delivered transendocardially ([32]). Although in a small trial, the early results are encouraging showing that the allogeneic cells increased ejection fraction and functional activity with no significant serious adverse events. The CONCERT-HF trial is an ongoing study investigating the combination of MSCs and c-kit^+^ cardiac stem cells in ischemic cardiomyopathy (NCT02501811), and is, interestingly, a trial that employs autologous bone marrow-derived MSCs. While the original approach for cell-based therapy in heart failure focused on using autologous cells, investigators are now using allogeneic cells because of the potential to deliver larger numbers of cells without harvesting from the patient. Allogeneic cells can be obtained from younger subjects where the cells may have more regenerative capacity based on protein expression and bioinformatics [3]. Furthermore, it has been suggested that HF, at its central process, is an inflammatory process [36]. In addition, it has been shown that MSCs are potently anti-inflammatory [5]. Knowing this, it follows that MSCs could slow and potentially reverse the ill effects of HF, if not too far progressed. Lastly, using allogeneic cells would be less costly and be able to deliver a true “off the shelf” approach. Allogeneic MSC therapy has been shown to be as safe and efficacious as autologous MSC therapy [32, 47, 49, 56, 69, 73, 75–78].

The primary mechanism by which MSCs are able to promote endogenous repair appears to be via paracrine signaling; however, the cells still need to hone to the area of interest and be retained, at least for some duration, to exert their therapeutic effects. Furthermore, the area(s) where MSCs hone to must have at least some vascularization given that MSCs act via paracrine signaling. Current administration of MSCs remains entrenched in traditional approaches including intravenous (IV) and intracardiac (IC) modes of delivery [34, 42–44, 79]. Recent trials have investigated the use of allogeneic MSCs, which has been safe and effective in a comparable capacity to autologous MSC therapeutic strategies [42]. Furthermore, preclinical studies as well as data from clinical trials have suggested that young donor MSCs have different signaling pathways activated when compared with older donor MSCs, given the changing cellular dynamics of aged MSCs [3, 44]. Thus, use of allogeneic young donor MSCs for treatment in an “off the shelf” therapeutic option make MSCs even more favorable as a cell-based therapy modality. This approach is being used in the Dream-HF trial with allogeneic mesenchymal precursor cells (NCT02032004). Indeed, an innovative delivery of MSCs could provide the missing component to propel MSCs as the long-sought-after treatment option for HF.

## 8. Delivery of MSCs with Biomaterials

Delivery with tissue-engineered biomaterials could provide an innovative delivery system to enable MSCs to further develop as a treatment option for HF and other cardiovascular disorders. Administration of MSCs via a venous approach has the risk of cells honing to an area of damage that is outside the heart. While IC administration of MSCs will confirm that MSCs will be present in the heart, often the cells are injected into the scar, which given its lack of adequate vascular supply, would not be hospitable to MSCs as therapeutics. Biomaterials are gaining increasing interest, especially with the advent of new technologies that allow for innovative treatment modalities. Administration of MSCs with the aid of biomaterials such as a scaffold could potentially resolve some of the perceived issues with MSC delivery in the setting of HF. Mesenchymal stem cells have been used in combination with biomaterials in preclinical studies with some promising results. Investigators injected a self-setting salinized hydroxypropyl methylcellulose seeded with MSCs and showed improvements in LV remodeling and infarct expansion in a rat model of myocardial infarction [80]. Another approach to improve retention of transplanted cells in the diseased heart is to inject the cells in an in situ cross-linked alginate hydrogel [81]. Adipose-derived MSCs embedded in alginate retain their viability, maintain their paracrine potential, and are not immunogenic suggesting that using alginate hydrogels may be a method to enhance delivery of MSCs in the clinical arena. A similar approach encapsulating MSCs in an alginate hydrogel patch has shown potential clinical benefit in a rat infarct model with evidence of improved cardiac function, decreased scar size, and increased peri-infarct vasculature [82].

The ideal scaffold would allow delivery of MSCs in such a way that would allow the cells to exert their paracrine signaling and not to impede the release of these secreted factors. Furthermore, the scaffold used for cell delivery would create not only a vector for delivery of the cells but also a more hospitable microenvironment from which MSCs could direct endogenous repair. It would also be advantageous if the scaffold could help generate its own new blood supply. This would overcome the current dilemmas of how to target MSCs to specific areas of the heart as well as concerns of engraftment in a potentially hostile environment, the postinfarction myocardium. Additionally, a biodegradable scaffold would exist only transiently; once the cells have established, the scaffold would no longer be needed. Among the current hypotheses of MSCs used as therapeutics, one suggested that MSCs typically initiate and direct the early phases of endogenous repair, and once the process is sufficiently underway, these cells are not required and are not retained for longer periods. Ideally, the scaffold would result in minimal inflammation, remain only transiently, not require removal, and promote angiogenesis.

## 9. Cellular Scaffolds

**Figure 2:**
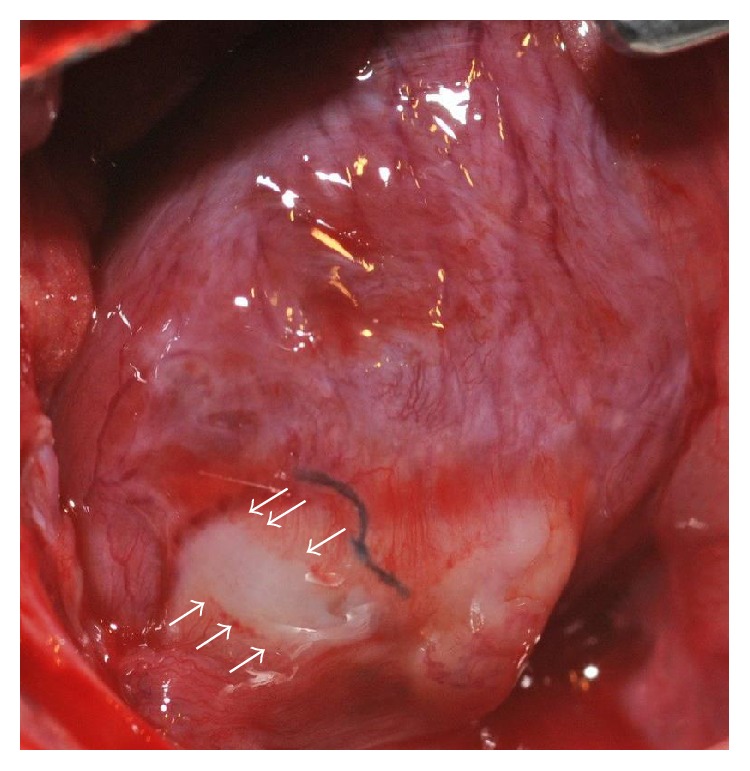
Cellular scaffold (arrows) placed over a scar in a rat heart *in vivo* 3 weeks after infarction. Three weeks after patch implantation, 6 weeks after infraction, evidence of angiogenesis from the patch to native myocardium was observed.

Several approaches have been suggested or are under investigation as cellular delivery alternatives to IV or IC administration of cells. Among these methods are cell- or tissue-based scaffold, electroshock-assisted cell delivery, and polymers for transport [83–86]. Criticisms of cell-based therapies point towards poor retention of cells, insufficient number of cells utilized for therapy, poor engraftment in the hostile conditions of HF, disruption of molecular honing signals, and excess extracellular matrix secondary to fibrosis as the pitfalls of cell administration-based therapies as options for HF. The potential pitfalls described in cell-based therapies can all be overcome by the use of cellular/tissue scaffolds. Previously, we have described a cellular scaffold that has been developed and demonstrated hemodynamic improvement as well as promotion of angiogenesis (Figure 2) [87, 88]. Such a cellular scaffold would not only allow for adequate delivery in regard to cell number but also location as it could be placed over the region of the scar. Furthermore, the scaffold would provide a more hospitable setting than the surrounding infarct, which would otherwise be much more hostile for MSC function. The scaffold would thus work in conjunction with MSCs to modulate the cellular microenvironment to make it more favorable and promote cellular repairs. MSCs administered with the scaffold would provide the necessary components for increased angiogenesis to occur, while reducing the need for honing and engraftment of MSCs. Utilizing a cellular scaffold, with known cardiovascular improvements in the setting of HF, with MSCs, given their potential as cellular therapeutics, could provide the elusive component necessary to progress cell-based therapies in HF.

## 10. Conclusion

MSCs were once touted as the ideal candidate for cell- and gene-based therapies. They were identified as the cells that would change regenerative medicine via their ability to differentiate into end-cell lines, allowing the shortage of donor organs to become a nonfactor in treatment of many end-stage disease states. As MSCs have been further investigated, it is their paracrine signaling that has come to the forefront and become the characteristic that makes them ideal candidates as cellular therapeutics. The ability to modulate the cellular microenvironment through expression of various cytokines and regulation of signal transduction pathways to direct and promote endogenous cellular repair is considered the hallmark function of MSCs. Furthermore, MSCs via their paracrine signaling are able to recruit dormant progenitor cells to aid in the regenerative process. Clinical trials have demonstrated that MSCs are safe and can play as a mainstay of treatment of HF. Novel delivery of MSCs as therapeutics in HF can overcome many of the current pitfalls such as hostile environment of HF for regenerative medicine and retention of cells. Cellular scaffolds in particular can assure that critical numbers of MSCs are able to reach the target area, whereby MSCs can then direct endogenous cellular repair.

## 11. Current and Future Perspectives

MSCs have been of interests for their potential as cellular therapeutics since their first description 37 years ago. Their ability to differentiate into end-cell lineages enticed investigators to believe that the dream of creating organs in the laboratory had become a reality. It was not until recently that it was determined that the therapeutic potential of MSCs was primarily in their mechanism of paracrine signaling, and not with the differentiation potentials. MSCs have been investigated in a myriad of disease states. Among the most devastating and costly disease of which currently remains is chronic HF. The scope of MSCs as potential therapies in HF is still in the very early stages. While significant progress is currently being made with ischemic HF clinical trials revealing that MSCs are not only safe for administration but may also provide the much anticipated therapeutic benefit. However, future research is needed to elucidate the ideal delivery of MSCs in the setting of ischemic cardiomyopathies, and research is needed in nonischemic cardiomyopathies as well. The mechanisms at play by which MSCs function to improve molecular and clinical state of HF need to be identified. Furthermore, clinically relevant endpoints of MSC therapy such as exercise time and functional capacity are important metrics to assess as we strive to improve the quality of life in patients with heart failure.

## Figures and Tables

**Table 1:** Clinical trials investigating mesenchymal stem cells in heart failure.

Study name	Trial	Phase	Results	Trial identifier	Citation
Comparison of allogeneic vs autologous bone marrow-derived mesenchymal stem cells delivered by transendocardial injection in patients with ischemic cardiomyopathy: the POSEIDON randomized trial (POSEIDON)	To test whether allogeneic MSCs are as safe and effective as autologous MSCs in patients with left ventricular (LV) dysfunction due to ICM	Phase 1/2 randomized	Patients with ICM that received transendocardial injection of allogeneic and autologous MSCs demonstrated favorable outcomes on patient functional capacity, quality of life, and ventricular remodeling without adverse effects	NCT01087996	[32]

Cardiopoietic stem cell therapy in heart failure: the C-CURE (cardiopoietic stem cell therapy in heart failure) multicenter randomized trial with lineage-specified biologics (C-CURE)	To evaluate the feasibility and safety of autologous bone marrow-derived and cardiogenically oriented mesenchymal stem cell therapy and to probe for signs of efficacy in patients with chronic heart failure	Prospective, multicenter, randomized trial	Cardiopoietic stem cell therapy was found feasible and safe with signs of benefit in chronic heart failure, meriting definitive clinical evaluation	NCT00810238	[67]

Transendocardial mesenchymal stem cells and mononuclear bone marrow cells for ischemic cardiomyopathy: the TAC-HFT randomized trial (TAC-HFT)	To demonstrate the safety of transendocardial stem cell injection with autologous mesenchymal stem cells (MSCs) and bone marrow mononuclear cells in patients with ischemic cardiomyopathy	Phase 1 and 2 randomized, blinded, placebo-controlledstudy	Transendocardial stem cell injection with MSCs or bone marrow mononuclear cells appeared to be safe for patients with chronic ischemic cardiomyopathy and LV dysfunction	NCT00768066	[73]

Rationale and design of the first randomized, double-blind, placebo-controlled trial of intramyocardial injection of autologous bone-marrow derived mesenchymal stromal cells in chronic ischemic heart failure (MSC-HF Trial) (MSC-HF)	To investigate the role of MSCs in patients with chronic ischemia utilizing intramyocardial injections in an ischemic viable region of the myocardium using the electromechanical NOGA-XP system	Phase 2, single-center, double-blind, randomized, placebo-controlledtrial	Intramyocardial injection of bone marrow-derived MSCs decreased LVEV, improved LV EF, increased stroke volume, and increased myocardial mass significantly compared to placebo	NCT00644410	[76]

A randomized study of transendocardial injection of autologous bone marrow mononuclear cells and cell function analysis in ischemic heart failure (FOCUS-HF) (FOCUS-HF)	To evaluate the safety and efficacy of the transendocardial delivery of autologous bone marrow mononuclear cells in patients with chronic HF	Phase 1, single-blind trial	Autologous bone marrow mononuclear cell therapy is safe and improves symptoms, quality of life, and possibly perfusion in patients with chronic HF	NCT00203203	[69]

RIMECARD	To assess the safety and efficacy of umbilical cord-derived MSCs in compensated dilated CM	Phase 1/2	Completed	NCT01739777	

TEVA CEP-41750 (DREAM-HF)	To evaluate efficacy and safety of allogeneic mesenchymal precursor cells (CEP-41750) for the treatment of chronic HF	Phase 3	Ongoing	NCT02032004	

Combination of mesenchymal and C-kit^+^ cardiac stem cells as regenerative therapy for heart failure (CONCERT-HF)	To evaluate the feasibility, safety, and effect of MSCs CSCs, and combination in heart failure of ischemic etiology, administered by transendocardial injection in ischemic cardiomyopathy	Phase 2 randomized, placebo-controlledclinical trial	Ongoing	NCT02501811	

Intravenous administration of allogeneic bone marrow-derived multipotent mesenchymal stromal cells (MSCs) in patients with recent onset anthracycline-associated cardiomyopathy	To learn if adding mesenchymal stem cells (MSCs) to standard-of-care drugs can help control heart failure that may have been caused by anthracyclines	Phase 1	Ongoing	NCT02408432	

Intravenous allogeneic mesenchymal stem cells for nonischemic cardiomyopathy: safety and efficacy results of a phase II-A randomized trial	To assess the safety and preliminary efficacy of intravenously administered ischemia-tolerant MSCs (itMSCs) in patients with nonischemic cardiomyopathy	Phase 2 single-blind, placebo-controlled, crossover, randomized	In patients with nonischemic cardiomyopathy, ischemia-tolerant MSC therapy was safe, caused immunomodulatory effects, and was associated with improvements in health status and functional capacity	NCT02467387	[72]

A randomized clinical trial of adipose-derived stem & regenerative cells in the treatment of patients with non revascularizable ischemic myocardium – the PRECISE trial (PRECISE)	To establish safety and feasibility of utilizing adipose derived stem & regenerative cells (ADRCs) in patients who have areas of myocardium that are not revascularizable and have demonstrated reversible ischemia	Phase 1, randomized, double-blind trial	Isolation and transendocardial injection of autologous ADRCs in no-option patients were safe and feasible	NCT00426868	

Allogeneic adipose tissue-derived stromal/stem cell therapy in patients with ischemic heart disease and heart failure - a safety study	To perform a small clinical safety trial in heart failure patients with allogeneic adipose tissue-derived mesenchymal stem cells	Phase 1	Ongoing	NCT02387723 2015-001560-19	

Stem cell therapy in ischemic non-treatable cardiac disease - SCIENCE A European Multi-Centre Trial (SCIENCE)	To investigate the efficacy of direct intramyocardial injection allogeneic adipose-derived stem cells in patients with reduced left ventricular ejection fraction (EF) (≤45%) and heart failure	Phase 2, double-blind, placebo-controlledtrial	Ongoing	NCT02673164 2015-002929-19	

Efficacy and safety of bone marrow-derived mesenchymal cardiopoietic cells (C3BS-CQR-1) for the treatment of chronic advanced ischemic heart failure (CHART-1)	To evaluate the safety and efficacy of C3BS-CQR-1 by comparing the overall response to standard of care and C3BS-CQR-1 relative to standard of care and a sham procedure	Phase 3, randomized, double-blind trial	Ongoing	NCT01768702	
